# Factors Associated With Risk of Postdischarge Thrombosis in Patients With COVID-19

**DOI:** 10.1001/jamanetworkopen.2021.35397

**Published:** 2021-11-22

**Authors:** Pin Li, Wei Zhao, Scott Kaatz, Katie Latack, Lonni Schultz, Laila Poisson

**Affiliations:** 1 Department of Public Health Sciences, Henry Ford Health System, Detroit, Michigan; 2 Department of Internal Medicine, Ascension St John Hospital, Detroit, Michigan; 3 Division of Hospital Medicine, Henry Ford Health System, Detroit, Michigan

## Abstract

**Importance:**

COVID-19 is associated with a high incidence of thrombotic events; however, the need for extended thromboprophylaxis after hospitalization remains unclear.

**Objective:**

To quantify the rate of postdischarge arterial and venous thromboembolism in patients with COVID-19, identify the factors associated with the risk of postdischarge venous thromboembolism, and evaluate the association of postdischarge anticoagulation use with venous thromboembolism incidence.

**Design, Setting, and Participants:**

This is a cohort study of adult patients hospitalized with COVID-19 confirmed by a positive SARS-CoV-2 test. Eligible patients were enrolled at 5 hospitals of the Henry Ford Health System from March 1 to November 30, 2020. Data analysis was performed from April to June 2021.

**Exposures:**

Anticoagulant therapy after discharge.

**Main Outcomes and Measures:**

New onset of symptomatic arterial and venous thromboembolic events within 90 days after discharge from the index admission for COVID-19 infection were identified using *International Statistical Classification of Diseases and Related Health Problems, Tenth Revision* codes.

**Results:**

In this cohort study of 2832 adult patients hospitalized with COVID-19, the mean (SD) age was 63.4 (16.7) years (IQR, 53-75 years), and 1347 patients (47.6%) were men. Thirty-six patients (1.3%) had postdischarge venous thromboembolic events (16 pulmonary embolism, 18 deep vein thrombosis, and 2 portal vein thrombosis). Fifteen (0.5%) postdischarge arterial thromboembolic events were observed (1 transient ischemic attack and 14 acute coronary syndrome). The risk of venous thromboembolism decreased with time (Mann-Kendall trend test, *P* < .001), with a median (IQR) time to event of 16 (7-43) days. There was no change in the risk of arterial thromboembolism with time (Mann-Kendall trend test, *P* = .37), with a median (IQR) time to event of 37 (10-63) days. Patients with a history of venous thromboembolism (odds ratio [OR], 3.24; 95% CI, 1.34-7.86), peak dimerized plasmin fragment D (D-dimer) level greater than 3 μg/mL (OR, 3.76; 95% CI, 1.86-7.57), and predischarge C-reactive protein level greater than 10 mg/dL (OR, 3.02; 95% CI, 1.45-6.29) were more likely to experience venous thromboembolism after discharge. Prescriptions for therapeutic anticoagulation at discharge were associated with reduced incidence of venous thromboembolism (OR, 0.18; 95% CI, 0.04-0.75; *P* = .02).

**Conclusions and Relevance:**

Although extended thromboprophylaxis in unselected patients with COVID-19 is not supported, these findings suggest that postdischarge anticoagulation may be considered for high-risk patients who have a history of venous thromboembolism, peak D-dimer level greater than 3 μg/mL, and predischarge C-reactive protein level greater than 10 mg/dL, if their bleeding risk is low.

## Key Points

**Question:**

Which patients with COVID-19 may benefit from extended thromboprophylaxis following hospital discharge?

**Findings:**

In this cohort study of 2832 patients hospitalized with COVID-19, postdischarge venous thromboembolic events occurred more often in those with a history of venous thromboembolism, peak dimerized plasmin fragment D (D-dimer) level greater than 3 μg/mL, and predischarge C-reactive protein level greater than 10 mg/dL. Patients who received postdischarge anticoagulation therapy had fewer events.

**Meaning:**

These findings suggest that postdischarge anticoagulation therapy may be considered for high-risk patients with COVID-19.

## Teaser

This cohort study identifies the factors associated with risk of postdischarge venous thromboembolism and evaluates the association of postdischarge anticoagulation use with venous thromboembolism incidence among patients with COVID-19.

## Introduction

COVID-19 induces coagulopathy manifested as elevation of dimerized plasmin fragment D (D-dimer) levels.^1,2,3,4,5^ As a result, patients with COVID-19 frequently experience both arterial thromboembolism (ATE) and venous thromboembolism (VTE). Bourguignon et al^6^ reported that pulmonary embolism and deep vein thrombosis occurred in 20.6% to 49.0% of patients with COVID-19 managed in intensive care units (ICUs). In many facilities, high-risk patients with COVID-19 are given anticoagulation (AC) at doses higher than the prophylactic dose for primary prophylaxis of VTE during their hospitalization.^7,8,9^ AC treatment in patients hospitalized with COVID-19 is associated with reduced mortality.^10,11,12^ Of note, the risk of ATE and VTE in patients with COVID-19 extends beyond their hospitalization. These thrombotic events are associated with readmission and mortality 90 days after discharge from the index admission. To mitigate the risk of VTE, a short-term course of AC has been used in patients with COVID-19 after their hospital discharge. This practice, however, is challenged by the low incidence of VTE in unselected patients with COVID-19. Giannis et al^13^ reported that 1.55% of patients with COVID-19 experienced VTE within 90 days after discharge. Universal prescription of postdischarge AC in patients with COVID-19 offers marginal clinical benefits and may cause harm in patients at high risk of bleeding. Given the unclear evidence, clinicians are facing the dilemma of which patients hospitalized with COVID-19 could benefit from postdischarge AC. We conducted a cohort study of patients with COVID-19 discharged from an inpatient hospital stay to assess the rate of postdischarge thrombosis in patients with COVID-19, identify the factors associated with the risk of postdischarge VTE, and evaluate the association of postdischarge AC use with VTE incidence.

## Methods

### Study Design

This analysis involved adult patients hospitalized with a diagnosis of COVID-19 confirmed by a positive polymerase chain reaction test at the 5 hospitals of Henry Ford Health System from March 1 to November 30, 2020. This study was approved by Henry Ford Health System institutional review board with a waiver of informed consent for retrospective medical record reviews of deidentified data, in accordance with 45 CFR §46. This study follows the Strengthening the Reporting of Observational Studies in Epidemiology (STROBE) reporting guidelines for cohort studies. Patients were excluded if they remained hospitalized at the time of analysis, died during hospitalization, or were discharged to hospice service. For patients with multiple admissions related to COVID-19, the first admission was considered as the index admission. The flowchart of patients’ enrollment is shown in eFigure 1 in the Supplement.

The first VTE or ATE events were identified up to 90 days after discharge from the index admission using the *International Statistical Classification of Diseases and Related Health Problems, Tenth Revision* codes listed in eAppendix 1 in the Supplement. Event and mortality rates were calculated at 90 days. Electronic records were queried by programmers to obtain patients’ demographic characteristics (age, sex, self-reported race of Black, White or other [ie, American Indian or Alaskan Native, Asian or Pacific Islander, any other race, or unknown], and body mass index), preexisting medical conditions, inpatient data (length of hospitalization and ICU admission), peak and predischarge laboratory results (C-reactive protein [CRP], D-dimer, absolute neutrophil count, absolute lymphocyte count, neutrophil lymphocyte ratio [NLR], platelet count, international normalization ratio, and partial thromboplastin time), use of AC during hospitalization and after discharge, and discharge places. Race was assessed in this study because Black patients have higher rates of incident VTE and pulmonary embolism than patients of other races.^14^ The accuracy of data was confirmed by manually reviewing patients’ medical records. The inpatient and discharge AC fulfilled are listed in eAppendix 2 in the Supplement. Up to 20% missingness was observed for selected laboratory results.

### Statistical Analysis

Descriptive statistics were calculated, with continuous variables described as mean (SD) or median (IQR). Categorical variables were described as frequency distributions. *t* test, Wilcoxon rank test, nonparametric Kruskal-Wallis, and Pearson χ^2^ test were used to compare variables by groups. Nonparametric Mann-Kendall trend test was used to test the monotonic trend of event number over time after discharge. The Cochran-Armitage trend test was used to test whether there was a linear trend of percentage of patients prescribed with postdischarge AC over time.

Univariable and multivariable logistic regression methods were used to assess factors associated with the risk of and medications for thrombosis. Multiple imputation with predictive mean matching was used to impute 5 complete data sets using the mice package in R.^15^ Forward selection with the log-likelihood ratio test as the criterion was used to select the factors potentially associated with risk of postdischarge VTE. The variables selected in 5 data sets with *P* < .05 were entered into the final multivariable model.

To evaluate the association of postdischarge AC use with new onset of VTE, propensity scores (PSs) were used to mitigate the confounding factors that influence postdischarge AC use and VTE events. Inverse PS weighting was used to assess the average treatment effect on the treated, which is defined as the effect of postdischarge AC (prophylactic or therapeutic) for those who received it, using the twang package in R.^16^ The twang methods rely on the generalized boosted model, a tree-based regression model, to calculate PSs that can avoid model misspecification and handle missing data in the variables. Standardized effect size and Kolmogorov-Smirnov statistics were used to assess the balance of the PS. Pairwise comparisons of PS for no treatment vs prophylactic AC and no treatment vs therapeutic AC are shown in eFigure 2 in the Supplement. The variables included in the PS model related to VTE were a history of VTE, peak neutrophil count, peak NLR, peak platelet count, peak and predischarge CRP greater than 10 mg/dL (to convert to milligrams per liter, multiply by 10), peak and predischarge D-dimer greater than 3 μg/mL (to convert to nanomoles per liter, multiply by 5.476), and peak and predischarge partial thromboplastin time longer than 35 seconds. The variables related to the postdischarge AC assignment were age, race, discharge place, inpatient length of stay, a history of ATE, a history of atrial fibrillation, predischarge neutrophil count, predischarge lymphocyte count, predischarge NLR, and discharge month. The Firth penalty was used to reduce the bias caused by rare events when estimating the average treatment effect on the treated.

All data were analyzed using SAS statistical software version 9.4 (SAS Institute) or R statistical software version 4.0.2 (R Project for Statistical Computing). Two-tailed *P* < .05 was the threshold for statistical significance. Data analysis was performed from April to June 2021.

## Results

### Events and Clinical Characteristics of Patients With COVID-19

In this cohort study of 2832 adult patients hospitalized with COVID-19, the mean (SD) age was 63.4 (16.7) years (IQR, 53-75 years), 1347 (47.6%) were male, 1102 (38.9%) were Black, and 1437 (50.7%) were White. Of these patients, 36 (1.3%) experienced new onset of VTE after discharge, among whom 16 had pulmonary embolism, 18 had deep vein thrombosis, and 2 had portal vein thrombosis. Fifteen patients (0.5%) with COVID-19 had a new onset of ATE after discharge, 1 had transient ischemic attack, and 14 had acute coronary syndrome. One hundred eight patients (3.8%) died within 90 days after discharge, and 6 died after thrombotic events, with 4 ATEs and 2 VTEs.

**Figure 1.**
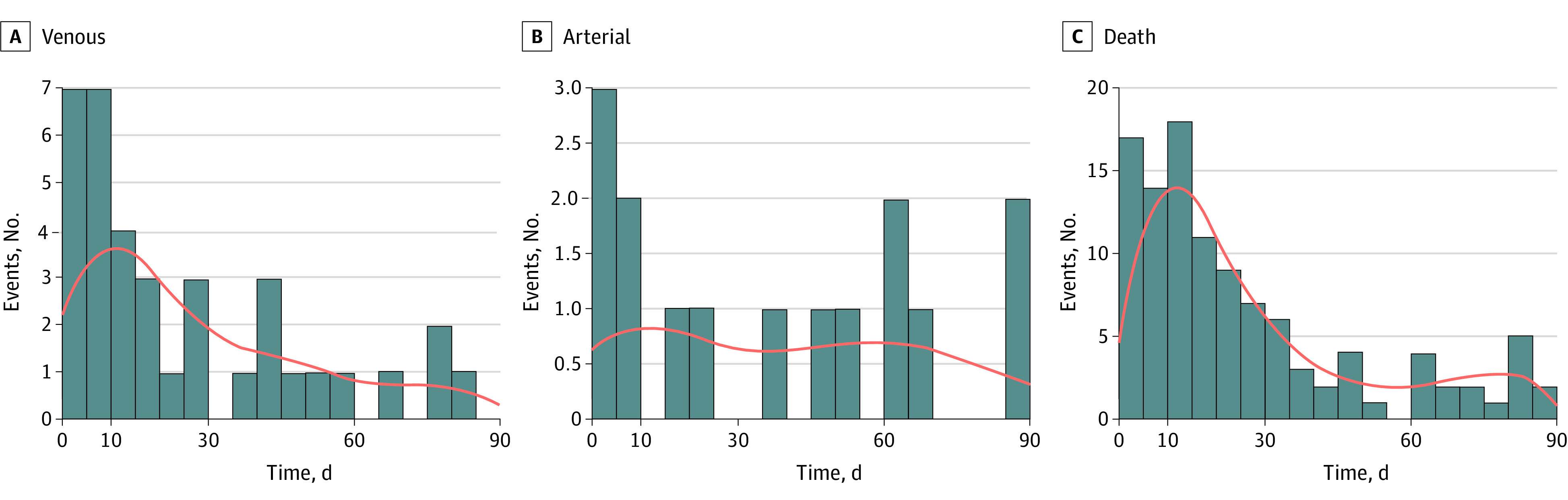
Event Counts of Postdischarge Venous Thromboembolism, Arterial Thromboembolism, and Death Without Events Orange lines denote kernel density.

The incidence of VTE decreased with time (Mann-Kendall trend test, *P* < .001), with a median (IQR) time to event of 16 (7-43) days (Figure 1). There was no change in the risk of ATE with time (Mann-Kendall trend test, *P* = .37), with the median (IQR) time to event of 37 (10-63) days. Mortality decreased with time (Mann-Kendall trend test, *P* < .001), with a median (IQR) time to death of 17 (9-34) days.

Clinical characteristics of our cohort are summarized in Table 1. Patients who experienced VTE after discharge more frequently had a history of VTE. Similarly, patients who experienced ATE after discharge more frequently had a history of atherosclerotic cardiovascular disease. Patients who died without events after discharge were older, had more comorbidities, and had a longer hospital stay.

**Table 1.** Demographic and Clinical Characteristics of Patients With COVID-19

Variables	Patients, No. (%)				*P* value
	No event (n = 2679)	VTE (n = 36)	ATE (n = 15)	Death (n = 102)	
Age at first admission, median (IQR), y	64.0 (52.0-75.0)	64.0 (58.8-75.0)	70.0 (59.0-74.0)	80.0 (70.0-87.0)	<.001^a^
Sex					
Female	1408 (52.6)	19 (52.8)	7 (46.7)	51 (50.0)	.93
Male	1271 (47.4)	17 (47.2)	8 (53.3)	51 (50.0)	
Race					
Black	1058 (39.5)	19 (52.8)	5 (33.3)	20 (19.6)	<.001^a^
White	1337 (49.9)	13 (36.1)	8 (53.3)	79 (77.5)	
Other^b^	284 (10.6)	4 (11.1)	2 (13.3)	3 (2.9)	
Discharge place					
Home	2117 (79.0)	25 (69.4)	9 (60.0)	53 (52.0)	<.001^a^
Nursing home	458 (17.1)	10 (27.8)	6 (40.0)	37 (36.3)	
Other hospital	52 (1.9)	0	0	9 (8.8)	
Rehabilitation facility	52 (1.9)	1 (2.8)	0	3 (2.9)	
Inpatient length of stay, median (IQR), d	6.0 (4.0-9.0)	6.0 (4.0-12.2)	6.0 (4.5-7.5)	7.0 (5.0-12.0)	.02^a^
Admitted to intensive care unit	406 (15.2)	7 (19.4)	2 (13.3)	15 (14.7)	.90
Body mass index, median (IQR)^c^	30.4 (26.2-36.3)	30.5 (27.8-36.1)	28.1 (22.5-34.5)	27.3 (23.3-32.3)	.001^a^
Medical history					
Deep vein thrombosis	128 (6.2)	4 (13.3)	1 (7.1)	5 (5.6)	.45
Pulmonary embolism	86 (4.2)	3 (10.0)	1 (7.1)	3 (3.3)	.40
Coronary artery disease	377 (18.3)	2 (6.7)	5 (35.7)	25 (27.8)	.01^a^
Myocardial infraction	193 (9.4)	1 (3.3)	1 (7.1)	16 (17.8)	.04^a^
Transient ischemic attack	264 (12.8)	4 (13.3)	3 (21.4)	14 (15.6)	.69
Hypertension	1747 (84.6)	24 (80.0)	14 (100.0)	77 (85.6)	.38
Diabetes	966 (46.8)	10 (33.3)	7 (50.0)	32 (35.6)	.09
Atrial fibrillation	221 (10.7)	2 (6.7)	1 (7.1)	23 (25.6)	<.001^a^
Cancer	391 (18.9)	8 (26.7)	1 (7.1)	27 (30.0)	.03^a^
Chronic kidney disease	303 (14.7)	7 (23.3)	5 (35.7)	11 (12.2)	.07
Laboratory values, median (IQR)					
Peak					
CRP, mg/dL	9.7 (4.3-15.8)	16.4 (9.4-23.1)	11.8 (3.7-19.4)	11.7 (5.8-19.1)	<.001^a^
D-dimer, μg/mL	1.4 (0.8-2.9)	3.5 (1.9-9.2)	1.6 (1.2-2.9)	2.3 (1.2-5.3)	<.001^a^
Neutrophils, cells/μL	8400 (5300-12 000)	11 900 (8500-15 500)	7900 (6200-15 100)	11 100 (7700-16 300)	<.001^a^
Lymphocyte, cells/μL	1300 (900-1900)	1.4 (900-2100)	1300 (900-2100)	1100 (700-1700)	.03^a^
NLR	11.1 (5.9-22.2)	19.9 (10.7-30.6)	17.4 (7.2-29.8)	23.0 (12.4-49.0)	<.001^a^
Platelet count, ×10^3^/μL	290 (220-390)	400 (290-450)	280 (250-330)	270 (200-370)	.005^a^
INR	1.1 (1.0-1.2)	1.2 (1.1-1.4)	1.2 (1.0-1.3)	1.3 (1.2-1.8)	<.001^a^
PTT, s	33.0 (29.0-39.0)	45.0 (33.5-112.5)	37.5 (31.2-44.5)	42.0 (32.0-89.0)	<.001^a^
Predischarge					
CRP, mg/dL	2.1 (0.9-5.8)	5.3 (0.7-11.3)	3.6 (1.3-7.1)	5.5 (2.3-11.5)	<.001^a^
D-dimer, μg/mL	1.0 (0.5-1.9)	2.3 (1.3-6.8)	1.2 (0.7-2.7)	1.7 (0.9-3.2)	<.001^a^
Neutrophils, cells/μL	5800 (3800-8500)	6600 (4900-9200)	6800 (4400-11 000)	8600 (5400-13 200)	<.001^a^
Lymphocyte, cells/μL	1100 (700-1500)	1000 (700-1500)	900 (600-1900)	700 (400-1100)	<.001^a^
NLR	5.3 (3.0-9.7)	5.6 (2.7-10.7)	4.8 (3.6-10.5)	12.7 (7.2-23.0)	<.001^a^
Platelet count, 10^3^/μL	260 (190-350)	320 (210-400)	260 (170-290)	200 (140-260)	<.001^a^
INR	1.1 (1.0-1.2)	1.1 (1.1-1.3)	1.1 (1.0-1.2)	1.2 (1.1-1.5)	<.001^a^
PTT, s	32.0 (29.0-37.0)	39.0 (30.5-78.5)	33.5 (30.2-39.8)	36.0 (31.0-49.0)	<.001^a^
Medication					
Inpatient AC					
No	304 (11.3)	2 (5.6)	2 (13.3)	6 (5.9)	<.001^a^
Prophylactic	1598 (59.6)	12 (33.3)	7 (46.7)	39 (38.2)	
Therapeutic	777 (29.0)	22 (61.1)	6 (40.0)	57 (55.9)	
Discharge					
Antiplatelets	868 (32.4)	8 (22.2)	8 (53.3)	27 (26.5)	.10
Statin	1110 (41.4)	11 (30.6)	7 (46.7)	41 (40.2)	.58
AC					
No	2038 (76.1)	34 (94.4)	10 (66.7)	68 (66.7)	<.001^a^
Prophylactic	174 (6.5)	0	4 (26.7)	10 (9.8)	
Therapeutic	467 (17.4)	2 (5.6)	1 (6.7)	24 (23.5)	

Abbreviations: AC, anticoagulation; ATE, arterial thromboembolism; CRP, C-reactive protein; D-dimer, dimerized plasmin fragment D; INR, international normalization ratio; NLR, neutrophil lymphocyte ratio; PTT, partial thromboplastin time; VTE, venous thromboembolism.

SI conversion factors: To convert CRP to milligrams per liter, multiply by 10; D-dimer to nanomoles per liter, multiply by 5.476; lymphocytes to 10^9^ per liter, multiply by 0.001; neutrophils to cells ×10^9^ per liter, multiply by 0.001; platelets to 10^9^ per liter, multiply by 1.

^a^ Denotes significance at *P* < .05 by nonparametric Kruskal-Wallis for continuous variables and Pearson χ^2^ test for categorical variables.

^b^ Other refers to American Indian or Alaskan Native, Asian or Pacific Islander, any other race, or unknown.

^c^ Body mass index is calculated as weight in kilograms divided by height in meters squared.

### Factors Associated With Risk of Postdischarge Thrombosis in Patients With COVID-19

Univariate analysis was performed to identify the factors associated with the risk of postdischarge VTE (Table 2). Postdischarge VTE was associated with a history of VTE (odds ratio [OR], 3.19; 95% CI, 1.35-7.52), peak platelet count (OR, 1.30; 95% CI, 1.07-1.58), peak (OR, 2.64; 95% CI, 1.26-5.52) and predischarge (OR, 3.67; 95% CI, 1.81-7.44) CRP levels greater than 10 mg/dL, peak absolute neutrophil count (OR, 1.07; 95% CI, 1.02-1.11), peak NLR (OR, 1.02; 95% CI, 1.01-1.03), peak (OR, 3.87; 95% CI, 1.95-7.66) and predischarge (OR, 4.21; 95% CI, 2.11-8.41) D-dimer levels greater than 3 μg/mL, and peak (OR, 3.11; 95% CI, 1.54-6.29) and predischarge (OR, 2.80; 95% CI, 1.43-5.50) partial thromboplastin time greater than 35 seconds.

**Table 2.** Univariable Analysis of the Factors Associated With Risk of Postdischarge VTE in Patients With COVID-19

Variables	Postdischarge VTE, participants, No. (%)		OR (95% CI)	*P* value
	No (n = 2796)	Yes (n = 36)		
Age at first admission, mean (SD), y	63.4 (16.8)	65.2 (13.7)	1.01 (0.99-1.03)	.50
Sex				
Female	1466 (98.7)	19 (1.3)	1 [Reference]	.97
Male	1330 (98.7)	17 (1.3)	0.99 (0.51-1.91)	
Race				
Black	1083 (98.3)	19 (1.7)	1 [Reference]	NA
White	1424 (99.1)	13 (0.9)	0.52 (0.26-1.06)	.07
Other^a^	289 (98.6)	4 (1.4)	0.79 (0.27-2.34)	.67
Discharge place				
Home	2179 (98.9)	25 (1.1)	1 [Reference]	NA
Nursing home	501 (98.0)	10 (2.0)	1.74 (0.83-3.65)	.14
Other hospital	61 (100.0)	0	0.00 (0.00-infinity)	.99
Rehabilitation facility	55 (98.2)	1 (1.8)	1.58 (0.21-11.91)	.66
Inpatient length of stay, mean (SD), d	7.9 (6.7)	8.6 (6.7)	1.01 (0.97-1.06)	.50
Intensive care unit admission				
No	2373 (98.8)	29 (1.2)	1 [Reference]	NA
Yes	423 (98.4)	7 (1.6)	1.35 (0.59-3.11)	.48
Body mass index, mean (SD)^b^	31.8 (8.5)	33.1 (10.8)	1.02 (0.98-1.05)	.37
Medical history				
History of VTE				
No	1979 (98.9)	23 (1.1)	1 [Reference]	NA
Yes	189 (96.4)	7 (3.6)	3.19 (1.35-7.52)	.008
History of atherosclerotic cardiovascular disease				
No	1523 (98.4)	25 (1.6)	1 [Reference]	NA
Yes	645 (99.2)	5 (0.8)	0.47 (0.18-1.24)	.13
Hypertension				
No	330 (98.2)	6 (1.8)	1 [Reference]	NA
Yes	1838 (98.7)	24 (1.3)	0.72 (0.29-1.77)	.47
Diabetes				
No	1163 (98.3)	20 (1.7)	1 [Reference]	NA
Yes	1005 (99.0)	10 (1.0)	0.58 (0.27-1.24)	.16
Atrial fibrillation				
No	1923 (98.6)	28 (1.4)	1 [Reference]	NA
Yes	245 (99.2)	2 (0.8)	0.56 (0.13-2.37)	.43
Cancer				
No	1749 (98.8)	22 (1.2)	1 [Reference]	NA
Yes	419 (98.1)	8 (1.9)	1.52 (0.67-3.43)	.32
Chronic kidney disease				
No	1849 (98.8)	23 (1.2)	1 [Reference]	NA
Yes	319 (97.9)	7 (2.1)	1.76 (0.75-4.15)	.19
Laboratory values				
Peak CRP >10 mg/dL				
No	1283 (99.2)	10 (0.8)	1 [Reference]	NA
Yes	1214 (98.0)	25 (2.0)	2.64 (1.26-5.52)	.01
Peak D-dimer >3 μg/mL				
No	1812 (99.2)	15 (0.8)	1 [Reference]	NA
Yes	593 (96.9)	19 (3.1)	3.87 (1.95-7.66)	<.001
Peak, mean (SD)				
Neutrophils, cells/μL	9400 (5500)	12 300 (5600)	1.07 (1.02-1.11)^c^	.002
Lymphocytes, cells/μL	1600 (2000)	1600 (900)	1.01 (0.90-1.14)^c^	.84
NLR	18.0 (19.5)	29.6 (28.7)	1.02 (1.01-1.03)	.001
Platelet count, 10^3^/μL	320 (140)	380 (130)	1.30 (1.07-1.58)^d^	.008
INR	1.3 (1.0)	1.4 (0.6)	1.09 (0.85-1.39)	.51
Peak PTT >35 s				
No	1415 (99.2)	12 (0.8)	1 [Reference]	NA
Yes	871 (97.4)	23 (2.6)	3.11 (1.54-6.29)	.002
Predischarge CRP >10 mg/dL				
No	2186 (99.0)	23 (1.0)	1 [Reference]	NA
Yes	311 (96.3)	12 (3.7)	3.67 (1.81-7.44)	<.001
Predischarge D-dimer >3 μg/mL				
No	2062 (99.0)	20 (1.0)	1 [Reference]	NA
Yes	343 (96.1)	14 (3.9)	4.21 (2.11-8.41)	<.001
Predischarge, mean (SD)				
Neutrophils, cells/μL	6700 (4000)	7100 (3600)	1.03 (0.95-1.10)^c^	.49
Lymphocyte, cells/μL	1200 (1100)	1200 (800)	0.98 (0.70-1.38)^c^	.93
NLR	8.4 (9.9)	10.9 (16.7)	1.02 (0.99-1.04)	.14
Platelet count, 10^3^/μL	280 (120)	310 (130)	1.19 (0.94-1.51)^d^	.16
INR	1.2 (0.5)	1.2 (0.4)	1.11 (0.63-1.96)	.72
Peak PTT >35 s				
No	1549 (99.0)	15 (1.0)	1 [Reference]	NA
Yes	737 (97.4)	20 (2.6)	2.80 (1.43-5.50)	.003
Medication				
Inpatient AC				
No	312 (99.4)	2 (0.6)	1 [Reference]	NA
Prophylaxis	1644 (99.3)	12 (0.7)	1.14 (0.25-5.11)	.87
Therapeutic	840 (97.4)	22 (2.6)	4.09 (0.96-17.47)	.06
Discharge antiplatelets				
No	1893 (98.5)	28 (1.5)	1 [Reference]	NA
Yes	903 (99.1)	8 (0.9)	0.60 (0.27-1.32)	.20
Discharge statin				
No	1638 (98.5)	25 (1.5)	1 [Reference]	NA
Yes	1158 (99.1)	11 (0.9)	0.62 (0.31-1.27)	.19
Discharge AC				
No	2116 (98.4)	34 (1.6)	1 [Reference]	NA
Prophylaxis	188 (100.0)	0	0.00 (0.00-infinity)	.98
Therapeutic	492 (99.6)	2 (0.4)	0.25 (0.06-1.06)	.06

Abbreviations: AC, anticoagulation; CRP, C-reactive protein; D-dimer, dimerized plasmin fragment D; INR, international normalization ratio; NA, not applicable; NLR, neutrophil lymphocyte ratio; OR, odds ratio; PTT, partial thromboplastin time; VTE, venous thromboembolism.

SI conversion factors: To convert CRP to milligrams per liter, multiply by 10; D-dimer to nanomoles per liter, multiply by 5.476; lymphocytes to 10^9^ per liter, multiply by 0.001; neutrophils to 10^9^ per liter, multiply by 0.001; platelets to 10^9^ per liter, multiply by 1.

^a^ Other refers to American Indian or Alaskan Native, Asian or Pacific Islander, any other race, or unknown.

^b^ Body mass index is calculated as weight in kilograms divided by height in meters squared.

^c^ OR is per increase of 1000 cells/μL.

^d^ OR is per increase of 10^5^ platelets/μL.

**Figure 2.**
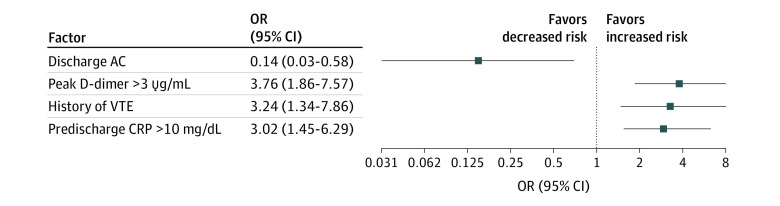
Multivariable Analysis of the Factors Associated With Risk of Postdischarge Venous Thromboembolism (VTE) in Patients With COVID-19 AC indicates anticoagulation; CRP, C-reactive protein; D-dimer, dimerized plasmin fragment D; OR, odds ratio.

Considering the correlations among factors associated with increased risk, results of the multivariable analysis showed that after adjusting for the other variables, patients with predischarge CRP levels greater than 10 mg/dL (OR, 3.02; 95% CI, 1.45-6.29), peak D-dimer levels greater than 3 μg/mL (OR, 3.76; 95% CI, 1.86-7.57), and a history of VTE (OR, 3.24; 95% CI, 1.34-7.86) were more likely to experience VTE after discharge. Postdischarge AC was associated with reduced risk of VTE after discharge (OR, 0.14; 95% CI, 0.03-0.58) (Figure 2).

### Postdischarge AC and VTE Incidence in Patients With COVID-19

Of our cohort, 682 patients (24.1%) received AC at discharge, with 188 patients (6.6%) receiving a prophylactic dose and 494 patients (17.4%) receiving a therapeutic dose. The percentage of patients prescribed with postdischarge AC was 8.6% (23 of 267 patients) in March 2020 (1.1% [3 patients] receiving a prophylactic dose and 7.5% [20 patients] receiving a therapeutic dose), and progressively increased to 35.5% (75 of 211 patients) in December 2020 (10.4% [22 patients] receiving a prophylactic dose and 25.1% [53 patients] receiving a therapeutic dose), with Cochran-Armitage trend test *P* < .001 (eFigure 3 in the Supplement). Patients who received inpatient therapeutic AC were more likely to receive postdischarge AC (OR, 27.2; 95% CI, 17.0-46.1; *P* < .001).

Patients who received the therapeutic AC at discharge had a reduced risk of experiencing new onset of VTE (OR, 0.18; 95% CI, 0.04-0.75; *P* = .02) (Table 3). The association of postdischarge prophylactic AC with postdischarge VTE was not significant after applying Firth penalty (OR, 0.17; 95% CI, 0.00-2.09; *P* = .29).

**Table 3.** Average Treatment Effect of Postdischarge AC to Prevent VTE in Patients With COVID-19^a^

Type of AC	OR (95% CI)	*P* value
Prophylactic AC	0.17 (0.00-2.09)	.26
Therapeutic AC	0.18 (0.04-0.75)	.02

Abbreviations: AC, anticoagulation; OR, odds ratio; VTE, venous thromboembolism.

^a^ The variables included in the propensity model related to VTE were a history of VTE, peak neutrophil count, peak neutrophil lymphocyte ratio, peak platelet count, peak and predischarge C-reactive protein greater than 10 mg/dL, peak and predischarge dimerized plasmin fragment D greater than 3 μg/mL, and peak and predischarge partial thromboplastin time longer than 35 seconds. The variables related to the postdischarge AC assignment were age, race, discharge place, inpatient length of stay, a history of arterial thromboembolism, a history of atrial fibrillation, predischarge neutrophil count, predischarge lymphocyte count, predischarge neutrophil lymphocyte ratio, and discharge month.

## Discussion

We conducted a cohort study involving 2832 adult patients hospitalized with COVID-19 to address the controversy regarding the use of AC after discharge. Our study has reiterated the low incidence of symptomatic VTE in patients with COVID-19 after discharge, which was comparable to other studies.^13,17,18,19,20,21,22^ To our knowledge, this study is the first so far that has captured enough postdischarge VTE events to be able to identify the factors associated with increased risk. We showed that patients hospitalized with COVID-19 having a history of VTE, predischarge CRP level greater than 10 mg/mL, or peak D-dimer level during hospitalization greater than 3 μg/mL were predisposed to experience new onset of VTE after discharge. Patients with these features were considered as a high-risk population. Postdischarge therapeutic AC was associated with reduced risk of VTE in all patients with COVID-19 requiring hospitalization. Because high-risk patients with COVID-19 had a higher incidence of VTE after discharge compared with other subpopulations, postdischarge therapeutic AC may benefit them the most.^20^ Our findings may help inform the future policy of postdischarge AC for patients hospitalized with COVID-19.

Prescription of AC for unselected patients hospitalized with COVID-19 at discharge is discouraged by a low incidence of symptomatic VTE and potential major bleeding complications. We reported that symptomatic VTE occurred in 1.3% of patients with COVID-19 up to 90 days after their discharge from hospital. The reported incidence of postdischarge thrombotic events in patients with COVID-19 varies across studies, in part because of the different lengths of follow-up, methods of follow-up, and postdischarge AC policies. Salisbury et al^17^ showed that the incidence of symptomatic VTE was 2.6% within 42 days of discharge in a cohort study of 303 patients from England. Eswaran et al^18^ found that 2.0% of 447 patients hospitalized with COVID-19 at University of North Carolina Health experienced a thromboembolic event within 30 days of discharge. A COVID registry study of 4906 patients from multiple health systems in New York State identified that the 90-day postdischarge VTE, ATE, and all-cause mortality rates were 1.55%, 1.71%, and 4.83%, respectively.^13^ It is safe to estimate that the incidence of VTE in recently discharged patients with COVID-19 is no more than 3% in the general population across the world. Importantly, postdischarge AC may increase bleeding. Giannis et al^13^ found that major bleeding rate was 2.45% in patients with COVID-19 who were discharged with AC, compared with 1.63% in those who were discharged without any AC. Our study further supports the National Institutes of Health COVID-19 treatment guideline,^23^ which recommends against routinely continuing AC for extended VTE prophylaxis after hospital discharge.

The International Medical Prevention Registry on Venous Thromboembolism (IMPROVE) tool and D-dimer level have been empirically incorporated in clinical decision-making to select high-risk patients hospitalized with COVID-19 who are most likely benefit from extended thromboprophylaxis.^23,24,25^ Postdischarge AC has been advocated for patients with COVID-19 who have an IMPROVE VTE risk score 4 or higher or an IMPROVE VTE risk score 2 or higher and D-dimer level more than 2 times the upper normal limit. The performance of IMPROVE tool in patients with COVID-19, however, remains unvalidated by data. Our study showed that the risk of experiencing new onset of symptomatic VTE after index hospitalization significantly increased in the subpopulation of our cohort who had a history of VTE, peak D-dimer greater than 3 μg/mL, and predischarge CRP greater than 10 mg/dL. Reliance on the IMPROVE tool to guide postdischarge AC in patients with COVID-19 should be done cautiously because 2 of 3 risk factors identified in this study are not reflected in the IMPROVE tool. Furthermore, the weighted risk factors in the IMPROVE tool, such as thrombophilia, active cancer, ICU stay and age, were not associated with postdischarge VTE in our cohort (Table 2). One possible explanation for this discrepancy is the different characteristics of our populations. We studied the adult patients hospitalized with COVID-19 during pandemic, whereas IMPROVE assessed acutely ill hospitalized medical patients before the pandemic. Furthermore, the pathogenesis of thrombosis in patients with COVID-19 may not be the same as that in general hospitalized patients. Also, we added laboratory data and use of anticoagulants to build the statistical model. Inclusion of these clinically important variables eliminated potential confounding variables. Giannis et al^13^ identified the factors associated with the risk of a composite outcome of ATE, VTE, and all-cause mortality, including advanced age, prior VTE, ICU stay, chronic kidney disease, peripheral arterial disease, carotid occlusive disease, IMPROVE D-dimer VTE score of 4 or higher, and coronary artery disease. Our risk factors are very different because of the different primary outcomes. The factors associated with increased risk of ATE, VTE, or all-cause mortality are not the same, as evidenced by the heterogeneity in patients’ characteristics across these groups (Table 1). We focused on postdischarge VTE as our primary outcome. Our findings are more relevant to help formulate an evidence-based postdischarge AC strategy for patients hospitalized with COVID-19.

Extending thromboprophylaxis beyond hospitalization using either prophylactic or therapeutic AC was not random in our cohort. Patients with COVID-19 considered at a high risk for VTE by physicians were more likely to receive postdischarge AC (eTable in the Supplement). As a result, conducting a multivariate analysis with limited variables is not sufficient to accurately evaluate the association of postdischarge AC with VTE incidence. By using a PS model to control the imbalances of patients’ characteristics that influenced the likelihood of receiving postdischarge AC, we found that patients with COVID-19 discharged with therapeutic AC were associated with a lower risk of symptomatic VTE (OR, 0.18; 95% CI, 0.04-0.75; *P* = .02). Of note, we did not distinguish dose level in this analysis and the strategy to choose the optimal AC dose needs further investigation.

### Limitations

This is a retrospective study that collected any re-encounters at the Henry Ford Health System main hospital and satellite hospitals after patients with COVID-19 were discharged up to 90 days from their index admission. No formal office or virtual follow-up was regularly scheduled for our cohort. We may underestimate the true incidence of symptomatic thrombotic events because patients might present to other hospitals outside of our network or might not present to any hospitals. Also, only 36 VTE and 18 ATE events were found in our cohort, significantly limiting our ability to build sophisticated multivariate models. Furthermore, certain categorical variables of interest are small groups, such as race and postdischarge prophylactic AC. This will limit modeling a rare disease. Finally, we did not assess the incidence of major bleeding attributed to postdischarge AC. Continuing postdischarge AC in high-risk patients with COVID-19 needs to be weighed against bleeding risks at clinicians’ discretion.

## Conclusions

In this cohort study of adult patients hospitalized with COVID-19, we found that patients who had a history of VTE, peak D-dimer greater than 3 μg/mL, and predischarge CRP greater than 10 mg/dL were at high risk of experiencing new onset of VTE after discharge from hospital. We propose that any patients with these risk factors be considered for postdischarge therapeutic AC if their bleeding risk is low.

## Notes

Supplement. **eFigure 1.** Flow Chart of Patients’ Enrollment **eAppendix 1.** *ICD-10* Codes for Arterial and Venous Thrombotic Events **eAppendix 2.** Inpatient and Discharge Anticoagulant List **eFigure 2.** Number of Patients With AC at Discharge by Month **eFigure 3.** Distribution of Propensity Scores by Treatment Group **eTable.** Univariable Analysis of the Predictors for Giving Extended Thromboprophylaxis to COVID-19 Patients at Discharge
