# Supplementary material for: Factors Associated With Risk of Postdischarge Thrombosis in Patients With COVID-19
Source: JAMA Netw Open. 2021 Nov 22;4(11):e2135397. doi: 10.1001/jamanetworkopen.2021.35397 (PMC8609408; doi:10.1001/jamanetworkopen.2021.35397)
Supplement: Supplement. — eFigure 1. Flow Chart of Patients’ Enrollment eAppendix 1. ICD-10 Codes for Arterial and Venous Thrombotic Events eAppendix 2. Inpatient and Discharge Anticoagulant List eFigure 2. Number of Patients With AC at Discharge by Month eFigure 3. Distribution of Propensity Scores by Treatment Group eTable. Univariable Analysis of the Predictors for Giving Extended Thromboprophylaxis to COVID-19 Patients at Discharge [file jamanetwopen-e2135397-s001.pdf]

Supplemental Online Content

Li P, Zhao W, Kaatz S, Latack K, Schultz L, Poisson L. Factors associated with risk of postdischarge thrombosis in patients with COVID-19. *JAMA Netw Open*. 2021;4(11):e2135397. doi:10.1001/jamanetworkopen.2021.35397

eFigure 1. Flow Chart of Patients' Enrollment

eAppendix 1. *ICD-10* Codes for Arterial and Venous Thrombotic Events

eAppendix 2. Inpatient and Discharge Anticoagulant List

eFigure 2. Number of Patients With AC at Discharge by Month

eFigure 3. Distribution of Propensity Scores by Treatment Group

eTable. Univariable Analysis of the Predictors for Giving Extended Thromboprophylaxis to COVID-19 Patients at Discharge

This supplemental material has been provided by the authors to give readers additional information about their work.

© 2021 Li P et al. *JAMA Network Open.*

eFigure 1: Flow Chart of Patients' Enrollment

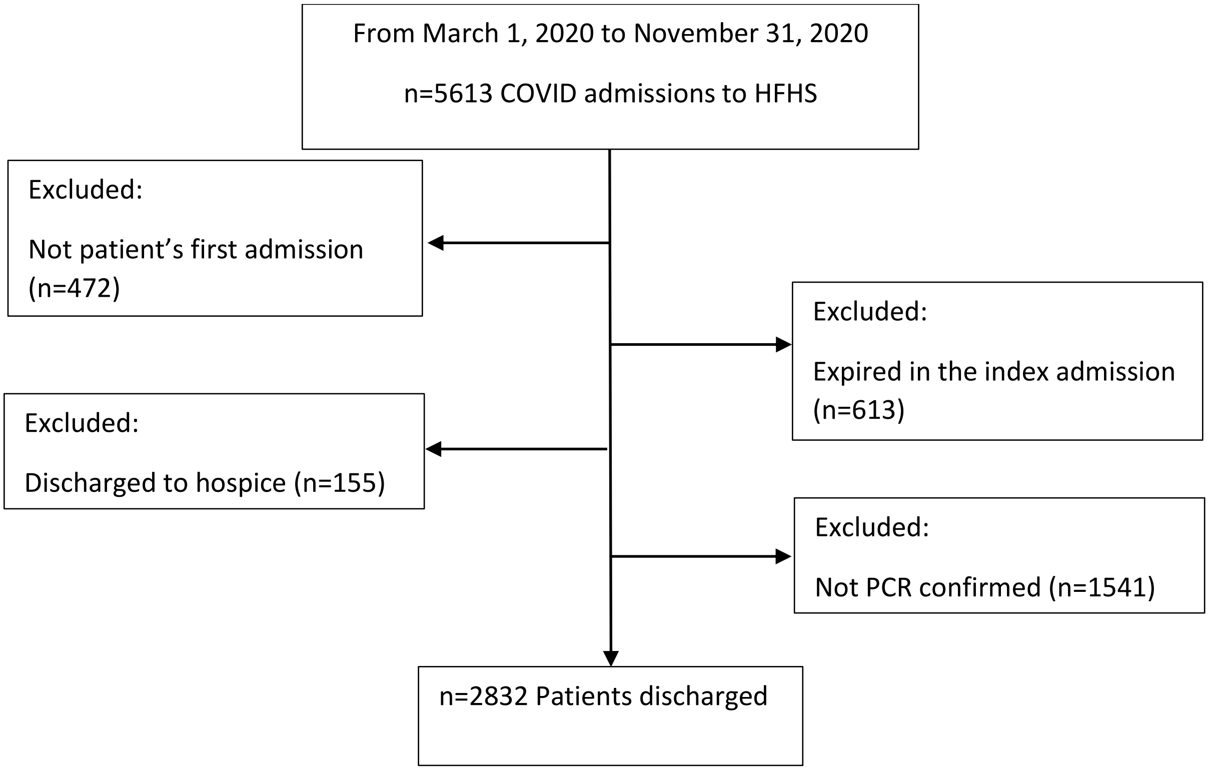

© 2021 Li P et al. *JAMA Network Open.*

eAppendix 1: *ICD-10* Codes for Arterial and Venous Thrombotic Events

Venous thrombosis:

I26 Pulmonary embolism

- I26.0 Pulmonary embolism with acute cor pulmonale
    - I26.02 Saddle embolus of pulmonary artery with acute cor pulmonale
    - I26.09 Other pulmonary embolism with acute cor pulmonale
- I26.9 Pulmonary embolism without acute cor pulmonale
    - I26.92 Saddle embolus of pulmonary artery without acute cor pulmonale
    - I26.93 Single subsegmental pulmonary embolism without acute cor pulmonale
    - I26.94 Multiple subsegmental pulmonary emboli without acute cor pulmonale
    - I26.99 Other pulmonary embolism without acute cor pulmonale

I67 Other cerebrovascular diseases

- I67.6 Nonpyogenic thrombosis of intracranial venous system

I81 Portal vein thrombosis

I82 Other venous embolism and thrombosis

- I82.0 Budd-Chiari syndrome
- I82.3 Embolism and thrombosis of renal vein
- I82.4 Acute embolism and thrombosis of deep veins of lower extremity
    - I82.40 Acute embolism and thrombosis of unspecified deep veins of lower extremity
        - I82.401 Acute embolism and thrombosis of unspecified deep veins of right lower extremity
        - I82.402 Acute embolism and thrombosis of unspecified deep veins of left lower extremity
        - I82.403 Acute embolism and thrombosis of unspecified deep veins of lower extremities, bilateral
        - I82.409 Acute embolism and thrombosis of unspecified deep veins of unspecified lower extremity
    - I82.41 Acute embolism and thrombosis of femoral vein
        - I82.411 Acute embolism and thrombosis of right femoral vein
        - I82.412 Acute embolism and thrombosis of left femoral vein
        - I82.413 Acute embolism and thrombosis of femoral veins, bilateral
        - I82.419 Acute embolism and thrombosis of unspecified femoral vein
    - I82.42 Acute embolism and thrombosis of iliac vein
        - I82.421 Acute embolism and thrombosis of right iliac vein
        - I82.422 Acute embolism and thrombosis of left iliac vein
        - I82.423 Acute embolism and thrombosis of iliac veins, bilateral
        - I82.429 Acute embolism and thrombosis of unspecified iliac vein
    - I82.43 Acute embolism and thrombosis of popliteal vein
        - I82.431 Acute embolism and thrombosis of right popliteal vein
        - I82.432 Acute embolism and thrombosis of left popliteal vein
        - I82.433 Acute embolism and thrombosis of popliteal veins, bilateral
        - I82.439 Acute embolism and thrombosis of unspecified popliteal vein
    - I82.44 Acute embolism and thrombosis of tibial vein
        - I82.441 Acute embolism and thrombosis of right tibial vein
        - I82.442 Acute embolism and thrombosis of left tibial vein
        - I82.443 Acute embolism and thrombosis of tibial veins, bilateral
        - I82.449 Acute embolism and thrombosis of unspecified tibial vein
    - I82.45 Acute embolism and thrombosis of peroneal vein
        - I82.451 Acute embolism and thrombosis of right peroneal vein
        - I82.452 Acute embolism and thrombosis of left peroneal vein
        - I82.453 Acute embolism and thrombosis of peroneal veins, bilateral

© 2021 Li P et al. *JAMA Network Open.*

        - I82.459 Acute embolism and thrombosis of unspecified peroneal vein
    - I82.46 Acute embolism and thrombosis of calf muscular vein
        - I82.461 Acute embolism and thrombosis of right calf muscular vein
        - I82.462 Acute embolism and thrombosis of left calf muscular vein
        - I82.463 Acute embolism and thrombosis of calf muscular veins, bilateral
        - I82.469 Acute embolism and thrombosis of unspecified calf muscular vein
    - I82.49 Acute embolism and thrombosis of other specified deep vein of lower extremity
        - I82.491 Acute embolism and thrombosis of other specified deep vein of right lower extremity
        - I82.492 Acute embolism and thrombosis of other specified deep vein of left lower extremity
        - I82.493 Acute embolism and thrombosis of other specified deep vein of lower extremities, bilateral
        - I82.499 Acute embolism and thrombosis of other specified deep vein of unspecified lower extremity
    - I82.4Y Acute embolism and thrombosis of unspecified deep veins of proximal lower extremity
        - I82.4Y1 Acute embolism and thrombosis of unspecified deep veins of right proximal lower extremity
        - I82.4Y2 Acute embolism and thrombosis of unspecified deep veins of left proximal lower extremity
        - I82.4Y3 Acute embolism and thrombosis of unspecified deep veins of proximal lower extremities, bilateral
        - I82.4Y9 Acute embolism and thrombosis of unspecified deep veins of unspecified proximal lower extremity
    - I82.4Z Acute embolism and thrombosis of unspecified deep veins of distal lower extremity
        - I82.4Z1 Acute embolism and thrombosis of unspecified deep veins of right distal lower extremity
        - I82.4Z2 Acute embolism and thrombosis of unspecified deep veins of left distal lower extremity
        - I82.4Z3 Acute embolism and thrombosis of unspecified deep veins of distal lower extremities, bilateral
        - I82.4Z9 Acute embolism and thrombosis of unspecified deep veins of unspecified distal lower extremity
- I82.6 Acute embolism and thrombosis of veins of upper extremity
    - I82.62 Acute embolism and thrombosis of deep veins of upper extremity
        - I82.621 Acute embolism and thrombosis of deep veins of right upper extremity
        - I82.622 Acute embolism and thrombosis of deep veins of left upper extremity
        - I82.623 Acute embolism and thrombosis of deep veins of upper extremities, bilateral
        - I82.629 Acute embolism and thrombosis of deep veins of unspecified upper extremity
- I82.A Embolism and thrombosis of axillary vein
    - I82.A1 Acute embolism and thrombosis of axillary vein
        - I82.A11 Acute embolism and thrombosis of right axillary vein
        - I82.A12 Acute embolism and thrombosis of left axillary vein
        - I82.A13 Acute embolism and thrombosis of axillary veins, bilateral
        - I82.A19 Acute embolism and thrombosis of unspecified axillary vein
- I82.B Embolism and thrombosis of subclavian vein
    - I82.B1 Acute embolism and thrombosis of subclavian vein
        - I82.B11 Acute embolism and thrombosis of right subclavian vein
        - I82.B12 Acute embolism and thrombosis of left subclavian vein
        - I82.B13 Acute embolism and thrombosis of subclavian veins, bilateral
        - I82.B19 Acute embolism and thrombosis of unspecified subclavian vein
- I82.8 Embolism and thrombosis of other specified veins
    - I82.81 Embolism and thrombosis of superficial veins of lower extremities
        - I82.811 Embolism and thrombosis of superficial veins of right lower extremity
        - I82.812 Embolism and thrombosis of superficial veins of left lower extremity
        - I82.813 Embolism and thrombosis of superficial veins of lower extremities, bilateral

© 2021 Li P et al. *JAMA Network Open.*

        - I82.819 Embolism and thrombosis of superficial veins of unspecified lower extremity

Arterial thrombosis:

G45 Transient cerebral ischemic attacks and related syndromes

- G45.0 Vertebro-basilar artery syndrome
- G45.1 Carotid artery syndrome (hemispheric)
- G45.2 Multiple and bilateral precerebral artery syndromes
- G45.3 Amaurosis fugax
- G45.4 Transient global amnesia
- G45.8 Other transient cerebral ischemic attacks and related syndromes
- G45.9 Transient cerebral ischemic attack, unspecified

I20 Angina pectoris

- I20.0 Unstable angina

I21 Acute myocardial infarction

- I21.0 ST elevation (STEMI) myocardial infarction of anterior wall
    - I21.01 ST elevation (STEMI) myocardial infarction involving left main coronary artery
    - I21.02 ST elevation (STEMI) myocardial infarction involving left anterior descending coronary artery
    - I21.09 ST elevation (STEMI) myocardial infarction involving other coronary artery of anterior wall
- I21.1 ST elevation (STEMI) myocardial infarction of inferior wall
    - I21.11 ST elevation (STEMI) myocardial infarction involving right coronary artery
    - I21.19 ST elevation (STEMI) myocardial infarction involving other coronary artery of inferior wall
- I21.2 ST elevation (STEMI) myocardial infarction of other sites
    - I21.21 ST elevation (STEMI) myocardial infarction involving left circumflex coronary artery
    - I21.29 ST elevation (STEMI) myocardial infarction involving other sites
- I21.3 ST elevation (STEMI) myocardial infarction of unspecified site
- I21.4 Non-ST elevation (NSTEMI) myocardial infarction
- I21.9 Acute myocardial infarction, unspecified

I22 Subsequent ST elevation (STEMI) and non-ST elevation (NSTEMI) myocardial infarction

- I22.0 Subsequent ST elevation (STEMI) myocardial infarction of anterior wall
- I22.1 Subsequent ST elevation (STEMI) myocardial infarction of inferior wall
- I22.2 Subsequent non-ST elevation (NSTEMI) myocardial infarction
- I22.8 Subsequent ST elevation (STEMI) myocardial infarction of other sites
- I22.9 Subsequent ST elevation (STEMI) myocardial infarction of unspecified site

I23 Certain current complications following ST elevation (STEMI) and non-ST elevation (NSTEMI) myocardial infarction (within the 28 day period)

- I23.6 Thrombosis of atrium, auricular appendage, and ventricle as current complications following acute myocardial infarction

I24 Other acute ischemic heart diseases

- I24.0 Acute coronary thrombosis not resulting in myocardial infarction
- I24.8 Other forms of acute ischemic heart disease
- I24.9 Acute ischemic heart disease, unspecified

I51.3 Intracardiac thrombosis, not elsewhere classified

I63 Cerebral infarction

- I63.0 Cerebral infarction due to thrombosis of precerebral arteries

  - I63.00 Cerebral infarction due to thrombosis of unspecified precerebral artery
  - I63.01 Cerebral infarction due to thrombosis of vertebral artery
    - I63.011 Cerebral infarction due to thrombosis of right vertebral artery
    - I63.012 Cerebral infarction due to thrombosis of left vertebral artery
    - I63.013 Cerebral infarction due to thrombosis of bilateral vertebral arteries
    - I63.019 Cerebral infarction due to thrombosis of unspecified vertebral artery
  - I63.02 Cerebral infarction due to thrombosis of basilar artery
  - I63.03 Cerebral infarction due to thrombosis of carotid artery
    - I63.031 Cerebral infarction due to thrombosis of right carotid artery
    - I63.032 Cerebral infarction due to thrombosis of left carotid artery
    - I63.033 Cerebral infarction due to thrombosis of bilateral carotid arteries
    - I63.039 Cerebral infarction due to thrombosis of unspecified carotid artery
  - I63.09 Cerebral infarction due to thrombosis of other precerebral artery
- I63.3 Cerebral infarction due to thrombosis of cerebral arteries
  - I63.30 Cerebral infarction due to thrombosis of unspecified cerebral artery
  - I63.31 Cerebral infarction due to thrombosis of middle cerebral artery
    - I63.311 Cerebral infarction due to thrombosis of right middle cerebral artery
    - I63.312 Cerebral infarction due to thrombosis of left middle cerebral artery
    - I63.313 Cerebral infarction due to thrombosis of bilateral middle cerebral arteries
    - I63.319 Cerebral infarction due to thrombosis of unspecified middle cerebral artery
  - I63.32 Cerebral infarction due to thrombosis of anterior cerebral artery
    - I63.321 Cerebral infarction due to thrombosis of right anterior cerebral artery
    - I63.322 Cerebral infarction due to thrombosis of left anterior cerebral artery
    - I63.323 Cerebral infarction due to thrombosis of bilateral anterior cerebral arteries
    - I63.329 Cerebral infarction due to thrombosis of unspecified anterior cerebral artery
  - I63.33 Cerebral infarction due to thrombosis of posterior cerebral artery
    - I63.331 Cerebral infarction due to thrombosis of right posterior cerebral artery
    - I63.332 Cerebral infarction due to thrombosis of left posterior cerebral artery
    - I63.333 Cerebral infarction due to thrombosis of bilateral posterior cerebral arteries
    - I63.339 Cerebral infarction due to thrombosis of unspecified posterior cerebral artery
  - I63.34 Cerebral infarction due to thrombosis of cerebellar artery
    - I63.341 Cerebral infarction due to thrombosis of right cerebellar artery
    - I63.342 Cerebral infarction due to thrombosis of left cerebellar artery
    - I63.343 Cerebral infarction due to thrombosis of bilateral cerebellar arteries
    - I63.349 Cerebral infarction due to thrombosis of unspecified cerebellar artery
  - I63.39 Cerebral infarction due to thrombosis of other cerebral artery
- I63.6 Cerebral infarction due to cerebral venous thrombosis, nonpyogenic

I74 Arterial embolism and thrombosis

- I74.2 Embolism and thrombosis of arteries of the upper extremities
- I74.3 Embolism and thrombosis of arteries of the lower extremities
- I74.4 Embolism and thrombosis of arteries of extremities, unspecified
- I74.5 Embolism and thrombosis of iliac artery

© 2021 Li P et al. *JAMA Network Open.*

eAppendix 2: Inpatient and Discharge Anticoagulant List

Inpatient anticoagulants:

Prophylaxis dose	Therapeutic dose
Enoxaparin 30 mg or 40 mg	Enoxaparin >40 mg
Heparin by subcutaneous	Heparin by intravenous
	Apixaban
	Argatroban
	Bivalirudin
	Dabigatran etexilate mesylate
	Edoxaban tosylate
	Fondaparinux sodium
	Rivaroxaban
	Warfarin sodium

Discharge medications:

Anti-platelets	Statin	AC prophylaxis dose	AC therapeutic dose
Aspirin	Atorvastatin	Apixaban 2.5 mg	Apixaban 5 mg or 10 mg
Clopidogrel	Lovastatin	Enoxaparin 30 mg or 40 mg	Enoxaparin >40 mg
Prasugrel	Pravastatin	Rivaroxaban 10 mg	Rivaroxaban 15 mg or 20 mg
Ticagrelor	Rosuvastatin		Dabigatran
	Simvastatin		Edoxaban
			Warfarin

© 2021 Li P et al. *JAMA Network Open.*

eFigure 2. Number of Patients With AC at Discharge by Month

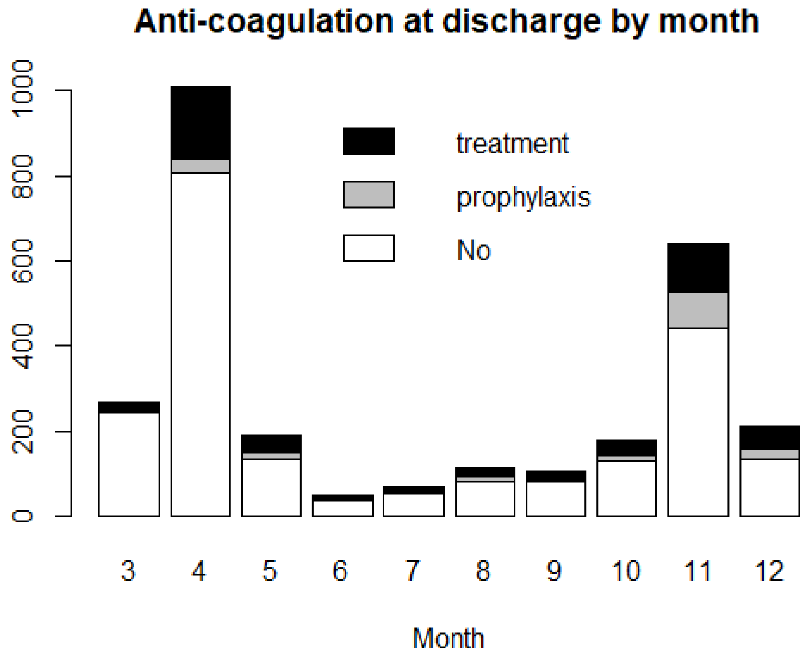

eFigure 3. Distribution of Propensity Scores by Treatment Group

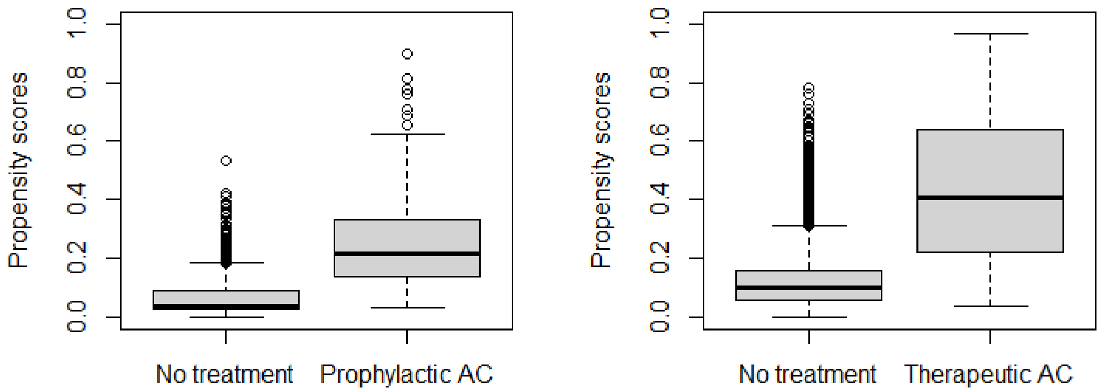

Pairwise comparisons are shown for “No treatment” vs “Prophylactic AC” (left), “No treatment” vs “Therapeutic AC” (right).

© 2021 Li P et al. *JAMA Network Open.*

eTable. Univariable Analysis of the Predictors for Giving Extended Thromboprophylaxis to COVID-19 Patients at Discharge

Variables		No (n=2150)	AC Prophylaxis (n=188)	AC Therapeutic (n=494)	Odds Ratio for AC (95% CI, p-value)
**Age at First Admission**	Mean (SD)	61.7 (17.1)	68.3 (16.0)	68.7 (13.7)	**1.03 (1.02-1.03, p<.001)**
Sex	Female	1137 (76.6)	112 (7.5)	236 (15.9)	-
	Male	1013 (75.2)	76 (5.6)	258 (19.2)	1.08 (0.91-1.28, p=.40)
**Race**	Black	911 (82.7)	41 (3.7)	150 (13.6)	-
	White	992 (69.0)	135 (9.4)	310 (21.6)	**2.14 (1.77-2.59, p<.001)**
	Other§	247 (84.3)	12 (4.1)	34 (11.6)	0.89 (0.63-1.25, p=.51)
**Discharge Place**	Home	1743 (79.1)	113 (5.1)	348 (15.8)	-
	Nursing home	330 (64.6)	63 (12.3)	118 (23.1)	**2.07 (1.68-2.55, p<.001)**
	Other hospital	31 (50.8)	10 (16.4)	20 (32.8)	**3.66 (2.19-6.11, p<.001)**
	Rehab	46 (82.1)	2 (3.6)	8 (14.3)	0.82 (0.41-1.64, p=.58)
**Inpatient Length of Stay**	Mean (SD)	7.3 (5.9)	9.3 (8.2)	10.0 (8.4)	**1.05 (1.04-1.06, p<.001)**
**ICU Admission**	No	1839 (76.6)	160 (6.7)	403 (16.8)	-
	Yes	311 (72.3)	28 (6.5)	91 (21.2)	1.25 (0.99-1.58, p=.06)
**BMI**	Mean (SD)	31.6 (8.4)	31.8 (9.4)	32.5 (8.8)	1.01 (1.00-1.02, p=.07)
**Medical History**					
**History VTE**	No	1515 (75.7)	139 (6.9)	348 (17.4)	-
	Yes	97 (49.5)	13 (6.6)	86 (43.9)	**3.18 (2.36-4.28, p<.001)**
**History ASCVD**	No	1176 (76.0)	100 (6.5)	272 (17.6)	-
	Yes	436 (67.1)	52 (8.0)	162 (24.9)	**1.55 (1.27-1.90, p<.001)**
**Hypertension**	No	250 (74.4)	20 (6.0)	66 (19.6)	-
	Yes	1362 (73.1)	132 (7.1)	368 (19.8)	1.07 (0.82-1.39, p=.63)
**Diabetes Mellitus**	No	864 (73.0)	89 (7.5)	230 (19.4)	-
	Yes	748 (73.7)	63 (6.2)	204 (20.1)	0.97 (0.80-1.17, p=.73)
**Atrial Fibrillation**	No	1523 (78.1)	131 (6.7)	297 (15.2)	-
	Yes	89 (36.0)	21 (8.5)	137 (55.5)	**6.32 (4.77-8.37, p<.001)**
**Cancer**	No	1301 (73.5)	124 (7.0)	346 (19.5)	-
	Yes	311 (72.8)	28 (6.6)	88 (20.6)	1.03 (0.81-1.31, p=.79)
**Chronic Kidney Disease**	No	1382 (73.8)	136 (7.3)	354 (18.9)	-
	Yes	230 (70.6)	16 (4.9)	80 (24.5)	1.18 (0.91-1.53, p=.22)
**Lab Values**					
**Peak CRP >10 mg/dL**	No	966 (74.7)	102 (7.9)	225 (17.4)	-
	Yes	923 (74.5)	76 (6.1)	240 (19.4)	1.01 (0.85-1.21, p=.90)
**Peak D-Dimer > 3 μg/mL**	No	1417 (77.6)	127 (7.0)	283 (15.5)	-
	Yes	399 (65.2)	46 (7.5)	167 (27.3)	**1.84 (1.51-2.25, p<.001)**
**Peak Neutrophils – k/μL**	Mean (SD)	9.0 (5.2)	9.9 (5.5)	10.8 (6.4)	**1.05 (1.03-1.06, p<.001)**
**Peak Lymphocyte – k/μL**	Mean (SD)	1.6 (2.2)	1.4 (0.8)	1.6 (1.1)	1.00 (0.96-1.04, p=.99)
**Peak NLR**	Mean (SD)	16.8 (18.3)	20.1 (20.7)	23.2 (24.0)	**1.01 (1.01-1.02, p<.001)**

© 2021 Li P et al. *JAMA Network Open.*

**Peak** Platelet Count – 10^5/µL	Mean (SD)	3.2 (1.4)	3.1 (1.2)	3.2 (1.4)	1.01 (0.95-1.08, p=.72)
**Peak INR**	Mean (SD)	1.2 (0.6)	1.2 (0.5)	1.9 (1.8)	**2.23 (1.90-2.62, p<.001)**
**Peak PTT > 35 sec**	No	1177 (82.5)	109 (7.6)	141 (9.9)	-
	Yes	519 (58.1)	52 (5.8)	323 (36.1)	**3.40 (2.81-4.12, p<.001)**
**Predischarge CRP >10 mg/dL**	No	1645 (74.5)	160 (7.2)	404 (18.3)	-
	Yes	244 (75.5)	18 (5.6)	61 (18.9)	0.94 (0.72-1.24, p=.68)
**Predischarge D-Dimer > 3 µg/mL**	No	1571 (75.5)	151 (7.3)	360 (17.3)	-
	Yes	245 (68.6)	22 (6.2)	90 (25.2)	**1.41 (1.10-1.80, p=.006)**
**Predischarge Neutrophils – k/µL**	Mean (SD)	6.5 (4.0)	7.1 (4.1)	7.1 (4.1)	**1.03 (1.01-1.05, p=.002)**
**Predischarge Lymphocyte – k/µL**	Mean (SD)	1.2 (1.1)	1.0 (0.6)	1.2 (0.9)	**0.89 (0.80-1.00, p=.05)**
**Predischarge NLR**	Mean (SD)	8.0 (9.4)	10.3 (13.1)	9.5 (11.1)	**1.02 (1.01-1.02, p<.001)**
Predischarge Platelet Count – 10^5/µL	Mean (SD)	2.8 (1.3)	2.6 (1.1)	2.7 (1.2)	**0.89 (0.83-0.96, p=.003)**
**Predischarge INR**	Mean (SD)	1.1 (0.4)	1.1 (0.2)	1.5 (0.8)	**4.58 (3.44-6.11, p<.001)**
**Predischarge PTT > 35 sec**	No	1267 (81.0)	119 (7.6)	178 (11.4)	-
	Yes	429 (56.7)	42 (5.5)	286 (37.8)	**3.26 (2.69-3.95, p<.001)**
**Medication**					
**Inpatient AC**	No	296 (94.3)	1 (0.3)	17 (5.4)	-
	Prophylaxis	1529 (92.3)	106 (6.4)	21 (1.3)	1.37 (0.82-2.27, p=.23)
	Therapeutic	325 (37.7)	81 (9.4)	456 (52.9)	**27.17 (16.56-44.59, p<.001)**
**Discharge Antiplatelets**	No	1457 (75.8)	124 (6.5)	340 (17.7)	-
	Yes	693 (76.1)	64 (7.0)	154 (16.9)	0.99 (0.82-1.19, p=.90)
**Discharge Statin**	No	1323 (79.6)	105 (6.3)	235 (14.1)	-
	Yes	827 (70.7)	83 (7.1)	259 (22.2)	**1.61 (1.35-1.91, p<.001)**

- Reference group for odds ratio
§ Other: American Indian or Alaskan Native, Asian or Pacific Islander, Other, Unknown

AC = anticoagulation, ASCVD = atherosclerotic cardiovascular disease, BMI = body mass index, CRP = C-reactive protein, ICU = intensive care unit, INR = international normalization ratio, NLR = neutrophil lymphocyte ratio, PTT = partial thromboplastin time, VTE = venous thromboembolism

© 2021 Li P et al. *JAMA Network Open.*
